# A Case of Pseudogout in an Adolescent on Isotretinoin

**DOI:** 10.1155/crrh/7179310

**Published:** 2026-02-13

**Authors:** Toshihide Kuroe, Tayyaba Wajih, Narihan Osman, Vivian Chang, Lily Q. Lew

**Affiliations:** ^1^ Department of Pediatrics, Flushing Hospital Medical Center, Flushing, 11355, New York, USA, flushinghospital.org

## Abstract

Pseudogout or calcium pyrophosphate dihydrate deposition disease rarely occurs in the young. Known risk factors for pseudogout include age, previous surgery, trauma, metabolic conditions, and medications. Isotretinoin, a retinoid frequently used to control acne vulgaris, is known to cause arthralgia, arthritis, and myalgia. We describe a case of an adolescent using isotretinoin who presented with acute left upper extremity pain and weakness. Birefringent calcium pyrophosphate dihydrate crystals were seen on synovial fluid analysis. The patient’s symptoms resolved after discontinuing isotretinoin. This is the first reported case of pseudogout in an adolescent on isotretinoin.

## 1. Introduction

Calcium pyrophosphate dihydrate crystal deposition disease causes an arthritis known as pseudogout [1]. The typical presentation includes sudden intense pain of one or more joints accompanied by swelling, warmth, erythema, and limited function. Calcium pyrophosphate dihydrate crystals within the articular cartilage initiate an inflammatory process that leads to symptoms that can imitate other arthritides [2]. The knee, wrist, hip, shoulder, and elbow joints are often affected. An accurate and timely diagnosis can be challenging without a biochemical test specific for pseudogout [3]. Birefringent calcium pyrophosphate dihydrate crystals identified on synovial fluid analysis and chondrocalcinosis on imaging studies confirm the diagnosis of pseudogout. Acute pseudogout occurs sporadically in advanced age and rarely in the young. A diagnosis of pseudogout in individuals younger than 60 years of age requires an investigation for secondary causes and hereditary arthropathies [4]. Isotretinoin, a retinoid used to treat acne vulgaris, can cause arthritis [5]. Limited data exist on isotretinoin and pseudogout. We report this case to highlight the importance of performing arthrocentesis in unexplained joint pain in an adolescent especially while on isotretinoin.

## 2. Case Report

A previously healthy 16‐year‐old South Asian female presented with left upper extremity pain and weakness of one‐day duration. She was experiencing numbness, tingling, swelling, and radiating pain from the wrist to the elbow upon awakening. She slept with her arm extended in her usual sleep position. There was no history of trauma, fever, chills, headache, slurred speech, change in vision, recent travel, or exposure to sick contacts. She took isotretinoin 40 mg orally twice a day (1.0 mg/kilogram/day) and prednisone 20 mg orally once a day (20–40 mg/day) for 10 weeks to treat acne vulgaris and hidradenitis suppurativa. No extended or immediate family member had an arthropathy. On physical examination, her weight was 76.2 kg, height was 174.0 cm, body mass index was 25.5 kg/meter^2^ (85^th^ percentile), blood pressure was 128/84 mm of mercury, pulse was 99 beats per minute, and temperature was 37.6 degrees Celsius. She had no facial asymmetry. An enlarged thyroid gland or neck mass was not appreciated. The extended left elbow and partially flexed wrist did not display warmth or erythema. The left‐hand swelling impeded the handgrip strength test. Supination elicited pain and the left elbow was tender to touch. There were no abnormalities of other joints. She was neurologically intact.

**FIGURE 1:**
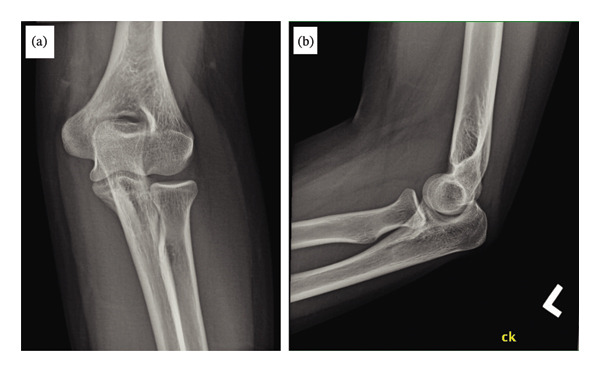
Chondrocalcinosis was absent on plain radiograph: (a) anteroposterior and (b) lateral views of the left elbow. Chondrocalcinosis or calcified cartilage is the deposition of calcium pyrophosphate dihydrate crystals within the articular cartilage.

**FIGURE 2:**
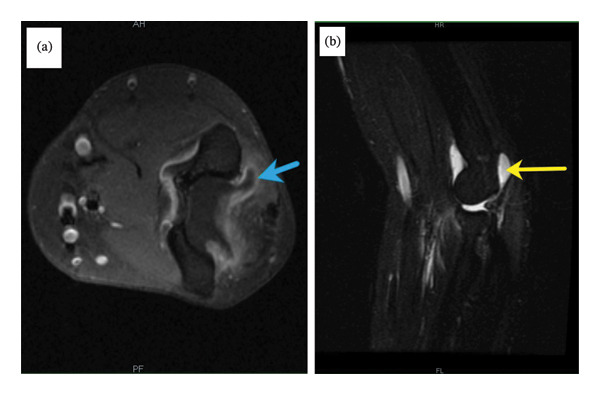
Magnetic resonance imaging of the left elbow demonstrated joint effusion with synovial enhancement on (a) T1‐weighted post gadolinium axial view (blue arrow) and (b) T2‐weighted post gadolinium sagittal view (yellow arrow). Arthrocentesis of the effusion provided the synovial fluid for analysis.

Laboratory test results are shown in Table 1. Plain radiograph of the left elbow did not demonstrate a fracture or chondrocalcinosis, as shown in Figures 1(a) and 1(b). Magnetic resonance imaging of the left elbow showed a large joint effusion, as shown in Figures 2(a) and 2(b). Aspirated synovial fluid from the left elbow was submitted for cell count and differential, Gram stain and culture. Polarized light microscopy revealed positively birefringent calcium pyrophosphate dihydrate crystals consistent with pseudogout. Birefringent negative monosodium urate crystals seen in gout were absent. After discontinuing isotretinoin, full range of motion returned with resolution of the hand swelling on the fourth hospital day. A nonsteroidal anti‐inflammatory drug was used for pain control during the hospitalization and as needed after hospital discharge.

**TABLE 1:** Laboratory findings.

Name of test (units)	Result	Reference range
Albumin (g/L)	44	34–54
Alkaline phosphatase (μkat/L)	2.02	0.62–2.10
Antinuclear antibody	Negative	Negative
Antistreptolysin O	Negative	Negative
Calcium (mmol/L)	2.48	2.10–2.55
C‐reactive protein (mg/L)	12.00	7.00–10.00
Creatine phosphokinase (U/L)	71.0	30.0–135.0
Epstein–Barr virus viral capsid antigen IgG (U/mL)	> 750.00	< 18.00
Epstein–Barr virus viral capsid antigen IgM (U/mL)	< 36.00	< 36.00
Erythrocyte sedimentation rate (mm/hour)	9	0–20
Ferritin (μg/L)	9.1	11–306.8
Hemoglobin (g/L)	110.00	120.00–160.00
Hemoglobin A1c (%)	5.4	< 5.7
Iron (μmol/L)	6.26	8.95–37.95
Lactate dehydrogenase (μkat/L)	6.46	5.23–10.32
Lyme disease antibody (index)	< 0.9	< 0.9
Magnesium (mmol/L)	0.82	0.78–1.11
Parathyroid hormone, intact (ng/L)	23.3	12.0–88.0
Parvovirus B19 IgG	0.1	< 0.9
Parvovirus B19 IgM	0.1	< 0.9
Phosphorus (mmol/L)	1.13	0.81–1.61
SARS‐CoV‐2 (polymerase chain reaction‐NP)	Negative	Negative
Synovial fluid		
Cell count: WBC/RBC (× 10^9^/L)	0.6/64.5	< 0.2–2.0/0–2.0
Gram stain	No organism	No organism
Culture	Negative	Negative
Thyroxine (nmol/L)	86.11	71.18–141.58
Thyroid‐stimulating hormone (mIU/L)	1.55	0.47–4.70
Total carbon dioxide (mmol/L)	26.00	22.00–30.00
Transferrin (μmol/L)	40.70	24.97–44.53
Uric acid (mmol/L)	0.21	0.14–0.39
Urinalysis	Negative	Negative
Vitamin D (25‐hydroxy vitamin D) (nmol/L)	32.5	74.9–249.6
White blood cell count (× 10^9^/L)	16.7	4.8–10.8
Neutrophils (× 10^9^/L)	13.7	1.8–7.0
Lymphocytes (× 10^9^/L)	1.8	1.2–3.7
Monocytes (× 10^9^/L)	0.7	0.2–1.2

*Note:* NP: nasopharygeal; SARS‐CoV‐2: severe acute respiratory syndrome 2.

Abbreviations: IgG, immunoglobulin G; IgM, immunoglobulin M; RBC, red blood cells; WBC, white blood cells.

## 3. Discussion

Although the 2011 European Alliance of Associations for Rheumatology (EULAR) consensus panel prefers the calcium pyrophosphate dihydrate crystal arthritis nomenclature, the term pseudogout persists to differentiate from the common form of gout [1]. Pseudogout and gout share similar clinical profiles but have dissimilar etiologies and therapeutic approaches. The estimated prevalence of pseudogout ranges from 4% to 7% of adults in the United States and Europe [2]. According to a recent review by Val et al., a definitive diagnosis of pseudogout remains dependent on demonstrating positive birefringence calcium pyrophosphate dihydrate crystals on synovial fluid analysis [3]. Since the risk of pseudogout increases with age, data are sparse on the prevalence in those aged less than 60 years [4]. The condition appears to be under‐recognized due to the wide range of presentations including being asymptomatic [6]. The American College of Rheumatology and EULAR drafted classification criteria for calcium pyrophosphate dihydrate deposition disease in 2022 to assist in the clinical diagnosis and treatment [7, 8].

Kohn et al. were the first to accurately identify crystalline material unlike the monosodium urate crystals seen in acute and chronic gout, as we now know as calcium pyrophosphate dihydrate crystals in 1962 [9]. The diagnosis requires the demonstration of birefringent calcium pyrophosphate dihydrate crystals by polarized light microscopy on synovial fluid analysis and chondrocalcinosis on plain radiograph [10]. The pathogenesis of pseudogout remains poorly understood. It is postulated that calcium pyrophosphate dihydrate crystals are formed as a complex of pyrophosphate and calcium generated by extracellular adenosine triphosphate (ATP). Modulators of extracellular ATP and pyrophosphate, and modulators of extracellular matrix factors regulate the formation of calcium pyrophosphate dihydrate crystals in articular cartilage with articular cartilage vesicles facilitating the crystal formation [11].

Underlying hypothyroidism, hyperparathyroidism, hypomagnesemia, and hemochromatosis as well as hereditary forms are known risk factors for pseudogout especially at younger ages [12]. Our patient had normal values for thyroid‐stimulating hormone, intact parathyroid hormone, serum magnesium, and transferrin. The low level of 25‐hydroxy vitamin D did not result in secondary hyperparathyroidism or change the serum calcium, phosphorus, and alkaline phosphatase levels. The presenting symptomology did not hint of a disturbance in vitamin D metabolism or a genetic cause of vitamin D deficiency. Improper nutrition, avoidance of sunlight exposure while on isotretinoin, and taking prescribed prednisone contributed to the low vitamin D. Hereditary pseudogout has been described as a result of a mutation in two genetic loci: long arm of Chromosome 8 (8q) and short arm of Chromosome 5 (5p) [13]. Additionally, pseudogout can occur in individuals with familial hypocalciuric hypercalcemia, an autosomal dominant disorder due to an inactivating mutation in the calcium‐sensing receptor gene on the long arm of Chromosome 3 (3q) in the majority [14, 15]. In the absence of hypercalcemia and hyperparathyroidism in our patient, determining urinary calcium excretion was not considered. Inquiry into genetic testing for these rare mutations was deferred in light of a negative family history. Moreover, there was no recent trauma or surgery, excluding post‐traumatic arthritis. A normal erythrocyte sedimentation rate and a negative antinuclear antibody test also exclude an autoimmune arthritis.

Of the crystalline arthropathies, gout and pseudogout occur most commonly [4]. Both gout and pseudogout represent inflammatory processes that have similar clinical presentations of sudden onset of pain with swelling, redness, and warmth. Unlike gout, pseudogout tends to affect large joints such as the knee. In gout, hyperuricemia and joint deposition of monosodium urate crystals are noted. Although pseudogout and gout can be present within the same joint [16], our patient had only one type of crystal in the synovial fluid and no evidence of hyperuricemia.

Presentation of pseudogout can simulate other arthritides, including rheumatoid arthritis, osteoarthritis, and septic arthritis. Both rheumatoid arthritis and osteoarthritis are typically seen in older individuals. The coexistence of rheumatoid arthritis and pseudogout plus rheumatoid arthritis and hidradenitis suppurativa has been described [17]. The findings on synovial fluid analysis and treatment are distinctly different when comparing pseudogout and septic arthritis [18]. The synovial fluid cell count and negative Gram stain in our afebrile patient did not support the diagnosis of septic arthritis and precluded antibiotic therapy. The leukocytosis was attributed to inflammation and high‐dose prednisone that persisted on follow‐up. Arthritides associated with Group A beta‐hemolytic streptococcus (poststreptococcal arthritis), Epstein–Barr virus (infectious mononucleosis), parvovirus B19 (gloves and socks syndrome), severe acute respiratory syndrome 2 or SARS‐CoV‐2 (viral arthritis), and *Borrelia burgdorferi* (Lyme disease) were excluded by their respective test.

Several medications have been implicated as the cause of pseudogout. Case reports have described the association of cancer therapeutic, loop diuretic, bisphosphonate, proton pump inhibitor, and tacrolimus with pseudogout [19–23]. Isotretinoin, a retinoic acid commonly used for the treatment of acne vulgaris, can cause arthralgia, myalgia, and arthritis [24, 25]. Retinoic acid has been identified as a modulator of extracellular ATP and pyrophosphate in the formation of calcium pyrophosphate dihydrate crystals. In a study by Rosenthal et al., increased inorganic pyrophosphate as seen in calcium pyrophosphate dihydrate deposition disease occurred with higher than natural levels of retinoic acid in porcine cartilage [11]. Our patient was not on the maximum dose of 2.0 mg/kilogram/day of isotretinoin but was on Week 10 of the higher recommended dose (0.5–1.0 mg/kilogram/day) since Week 5. Her calculated cumulative dose of isotretinoin was 4800 mg that included the starting dose of 0.5 mg/kilogram/day for Weeks 1‐4 followed by 1.0 mg/kilogram/day for Weeks 5–10. We linked the higher recommended dose of isotretinoin to an increase in inorganic pyrophosphate and to the formation of calcium pyrophosphate dihydrate crystals. Prednisone is prescribed commonly in conjunction with isotretinoin for its anti‐inflammatory property. There are no known reports of prednisone triggering arthritis or pseudogout.

We conclude that our patient had acute pseudogout while taking isotretinoin based on temporal relationship of medication use and its onset of symptoms. The known risk factors for pseudogout in the young were excluded. Other reported cases of pseudogout in an adolescent on isotretinoin do not exist. Our patient illustrated the importance of synovial fluid analysis to diagnose the etiology of joint pain. Clinician should be aware of the potential risk of pseudogout when on isotretinoin.

## Funding

No funding was received for this manuscript.

## Consent

A written informed consent was obtained from the patient for publication of the case report and accompanying images.

## Conflicts of Interest

The authors declare no conflicts of interest.

## Data Availability

The data used to support the findings of this study are included within the article.
